# *ASXL1* mutation-related clonal hematopoiesis and age-related diseases: clinical evidence and molecular insights

**DOI:** 10.1007/s12185-025-04038-5

**Published:** 2025-08-07

**Authors:** Naru Sato, Susumu Goyama, Toshio Kitamura

**Affiliations:** 1 Division of Molecular Oncology, Department of Computational Biology and Medical Sciences, Graduate School of Frontier Sciences, The University of Tokyo, Tokyo, Japan; 2 Molecular Pharmacology of Malignant Diseases, Graduate School of Pharmaceutical Sciences, The University of Tokyo, Tokyo, Japan; 3 Institute of Biomedical Research Innovation, Foundation for Biomedical Research Innovation at Kobe, Kobe, Japan

**Keywords:** ASXL1, clonal hematopoiesis, chronic inflammation,, autoimmune disease

## Abstract

Clonal hematopoiesis (CH) is defined as the age-associated expansion of hematopoietic stem and progenitor cells harboring somatic mutations, most frequently in epigenetic regulators such as *DNMT3A*, *TET2*, and *ASXL1*. Although CH was initially recognized as a precursor to hematological malignancies, accumulating evidence has led to its broad recognition as a relevant factor in various age-related nonmalignant diseases, particularly those with inflammatory components, such as cardiovascular disease, autoimmune disorders, and solid tumors. Notably, the increased overall mortality associated with CH is primarily driven by cardiovascular complications rather than hematological malignancies. Among CH-associated genes, *ASXL1* mutations are distinguished by their strong associations with adverse clinical outcomes and pro-inflammatory signatures. However, compared to *TET2* and *DNMT3A*, the molecular and pathological implications of *ASXL1*-mutated CH remain underexplored. Recent studies have expanded the disease spectrum of *ASXL1* mutations beyond hematological malignancies, implicating them in clonal expansion and systemic inflammation. This review aims to summarize the current epidemiological and experimental insights into *ASXL1*-mutated CH, focusing on its potential contributions to inflammation-associated diseases. By integrating clinical observations and emerging mechanistic data, we highlight the urgent need for deeper investigation into *ASXL1*-driven CH and its systemic consequences beyond hematological transformation.

## Introduction

Aging is commonly associated with various human diseases, such as solid cancers, chronic heart failure, atherosclerosis, and autoimmune diseases, and is traditionally viewed as a non-modifiable risk factor. Most tissues accumulate somatic mutations in a way that increases with age. In 2014, three groups examined exome sequencing data from studies that together comprised more than 30000 persons for the presence of mutations in peripheral blood cells [1–3]. They reported that clonal expansion of blood cells harboring somatic mutations associated with hematological malignancies, named clonal hematopoiesis (CH), increased in frequency and variant allele fraction (VAF) with aging, was present in about 10% of healthy individuals older than 65 years of age without diagnostic criteria for hematological abnormality. In this review, we use the term CH rather than clonal hematopoiesis of indeterminate potential (CHIP), as CHIP is defined by a VAF > 2% and by the absence of hematological disease, whereas our discussion includes ASXL1-mutated clones across a broader range of VAFs and clinical contexts.[4]. Because CH-related mutations can also be detected in circulating immune cells, this finding raises the possibility that CH may cause altered immune responses, potentially influencing many aging-related diseases.

Mutations in CH clones are thought to confer the fitness of hematopoietic stem cells (HSCs) and cause clonal expansion. However, mutations in classical oncogenes and tumor suppressors, such as those involved in cellular growth signaling (*JAK2*, *GNAS*, *GNB1*, *CBL*) and the DNA damage response (*TP53*, *PPM1D*), were rather rare. Instead, the most frequently mutated genes in individuals with CH were *DNMT3A*, *TET2*, and *ASXL1*, known as epigenetic regulators. These top 3 genes occupy more than 70% of the mutated genes in CH.

Several studies have found that CH is associated with a 30 to 40% increased mortality risk, and individuals with CH develop hematological malignancies at a higher rate than those without mutations [1, 3]. However, this risk of an increase in mortality could not be explained by cancer deaths but was instead related to increased cardiovascular disease (CVD) mortality. Further studies in additional cohorts reported that the risk for coronary heart disease was nearly twice as high for middle-aged and older individuals with CH compared to individuals without CH [5]. Moreover, emerging data also associate the presence of such clones with an increased risk of other non-hematological diseases: type 2 diabetes [1, 6], inflammatory bowel diseases (IBD) [7], gout [8], chronic liver diseases [9], autoimmune diseases [10], and solid cancers [11, 12]. Recent evidence also suggests the correlation between CH and non-myeloid hematological disorders: aplastic anemia (AA) [13, 14], large granular lymphocytic leukemia (LGLL) [15], and inflammatory Waldenström macroglobulinemia [16].

To clarify the causal relationship between CH and diseases, subsequent studies have extensively investigated CH with *TET2* or *DNMT3A* mutations. Numerous studies have demonstrated that mutations in these genes alter the immunological/inflammatory properties of blood cells, thereby increasing the risk of CVD. *Tet2* deficiency in bone marrow cells (BMCs) led to an increase of NLRP3 inflammasome-mediated interleukin (IL)-1β secretion in macrophages and a marked increase in atherosclerotic plaque size [5, 17]. Concerning the loss of *Dnmt3a*, it was originally reported that *Dnmt3a*-knockout (KO) BMCs worsened heart function through an increased accumulation of macrophages and other immune cells in the myocardium in experimental heart failure [18]. Another group reported that *Dnmt3a*-KO BMCs enhanced atherosclerosis like *Tet2*-KO BMCs [19]. *TET2*-CH is associated with increased IL-1β in humans, whereas CH with other driver mutations was not [20]. Considering that LPS-stimulated Dnmt3a^R878H/+^ neutrophils and monocytes released higher amounts of IL-1b, IL-6, and TNF than their Dnmt3a^+/+^ counterparts in vitro [21], *DNMT3A*-CH has the potential for increasing the inflammatory state, like *TET2*-CH. Indeed, the circulating monocytes derived from patients with heart failure harboring *DNMT3A* mutations showed significantly higher expression of *IL-1b*, *IL6*, and *CXCL2 *[22].

On the other hand, despite robust clinical connections of *ASXL1*-CH to CVD and other diseases, little is known about the causal effect of *ASXL1*-CH on them. This review provides an overview of the latest studies on *ASXL1*-CH and the relationship between *ASXL1*-CH and non-hematological diseases.

## *ASXL1* mutations in clonal hematopoiesis

CH-related gene mutations are similar to those in myeloid malignancies. Somatic mutations in Additional sex comb-like 1(*ASXL1*) are frequently detected in various types of myeloid malignancies, including myelodysplastic syndromes (MDS, 14–23%) [23–29], chronic myelomonocytic leukemia (CMML, 40–49%) [23, 30], myeloproliferative neoplasms (MPN, 5–11%) [31–34], and acute myeloid leukemia (AML, 5–17%) [24, 35, 36]. *ASXL1* mutations are related to the poor prognosis of these myeloid malignancies. Given that *ASXL1* mutations are detected broadly in various diseases and individuals with CH, *ASXL1* mutation is indicated to be one of the earliest genetic events during the process of myeloid transformation. Indeed, Triviai et al. reported that the mutations in *ASXL1* and/or *EZH2* were identified as the first genetic lesions in myelofibrosis (MF, 38%), preceding both *JAK2-V617F* and *CALR* mutations [37].

**Fig. 1:**
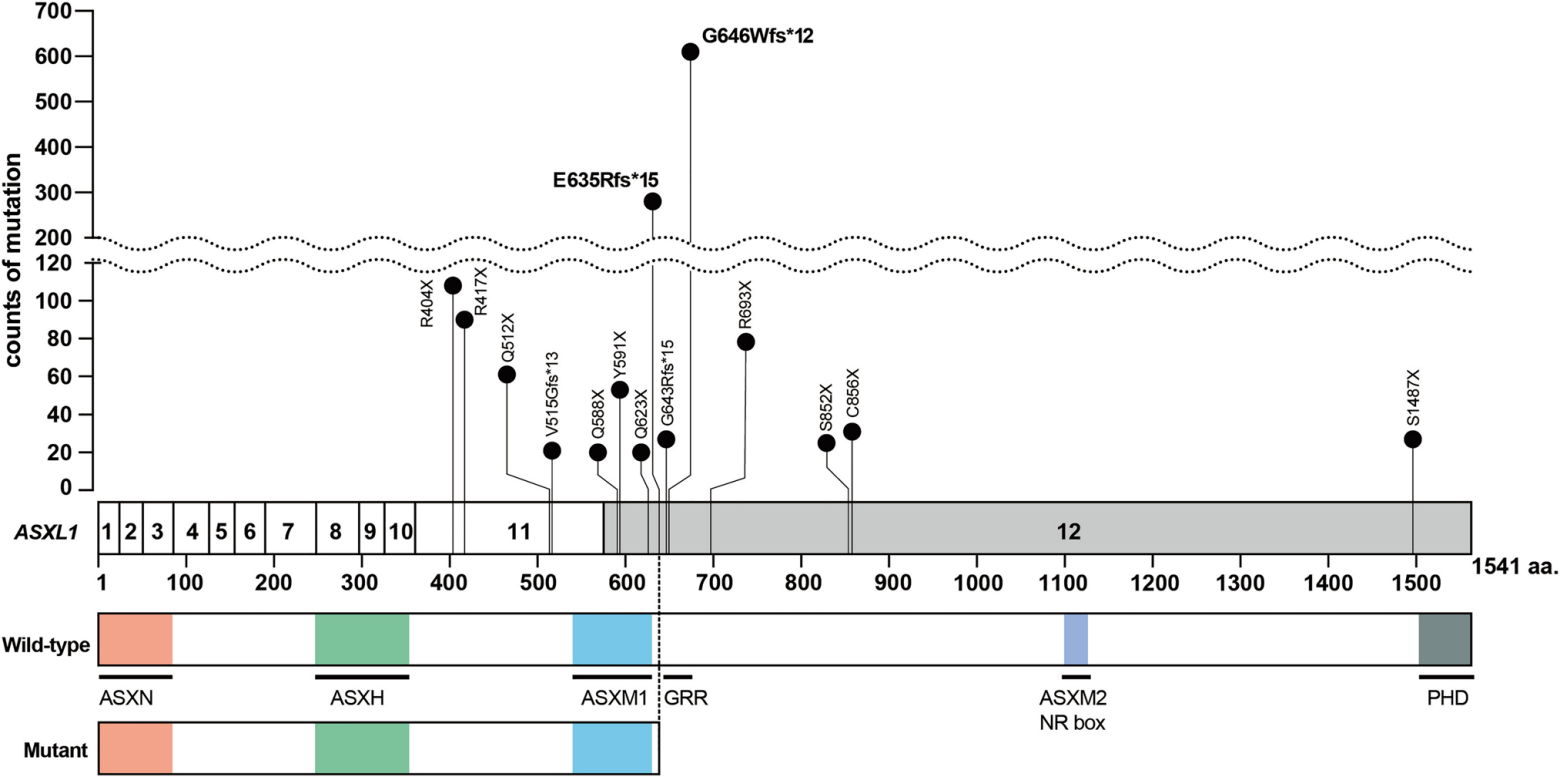
Localization of *ASXL1* mutations in individuals with CH modified from a previous study by Vlasschaert et al. with the UK biobank cohort [41]. Nonsense or frameshift mutations in the *ASXL1* gene are concentrated in the N-terminus of the last exon (gray colored), generating a C-terminally truncated form of ASXL1 (Mutant ASXL1). Hotspot mutations are indicated in boldface. *ASXH =* Asx homology; *ASXN* = Asx N-terminal; *GRR = *Glycine-rich region *NR* box = nuclear receptor co-regulator binding motif; *PHD* = plant homeodomain

*ASXL1* mutations are most often heterozygous frameshift or nonsense mutations in exon 12, producing a truncated protein. [38] The most common *ASXL1* mutation in myeloid neoplasms is *ASXL1* c.1934dupG (*ASXL1* NM_015338.5: c.1934dup; p.G646Wfs*12), accounting for approximately half of somatic truncating mutations. This duplication of a single guanine occurs within an eight-guanine nucleotide repeat that extends from c.1927 to c.1934, resulting in a truncated ASXL1 protein lacking a plant homeodomain (PHD) finger. *ASXL1* p.G646Wfs*12 was initially thought to represent a mere sequencing artifact by slipped-strand mispairing of the DNA polymerase during PCR amplification, but was later confirmed to be a bona fide mutation. Although this artifact may occur, subsequent Sanger sequencing reports identified the c.1934dup mutation in blood or marrow from patients but not in matched germline samples [38] or healthy controls [29]. More recently, it was reported that this mutation could also be identified by quantitative PCR [39]. Montes-Moreno et al. identified that sequencing methods that used PCR-only amplification introduced in vitro indels at the homopolymer locus encoding G645 and G646, whereas protocols using probe capture before PCR, as well as Sanger sequencing, did not [40]. When these sequencing methods are compared, p.G646Wfs*12 variants with a VAF ≥ 10% were true somatic variants, whereas all G645Vfs*58 were artifactual [40]. Using a higher VAF threshold for *ASXL1* p.G646Wfs*12 and removing p.G645Vfs*58 variants, Vlasschaert et al. analyzed ascertain CH in ~ 550,000 individuals [41]. They found that p.G646Wfs*12 variants with VAF ≥ 10% were associated with age, and the most frequent *ASXL1* mutation was also in CH, as in myeloid malignancies (Fig. 1). Of note, in this study, *ASXL1* p.G646Wfs*12 was the 3rd most common gene mutation in all CH mutations. Therefore, when interpreting cohort studies of somatic mutations in hematopoietic cells, it is important to verify whether *ASXL1* p.G646Wfs*12 was included or excluded in the analysis. In the UK Biobank cohort, the two most common *ASXL1* mutations detected in CH were p.G646Wfs*12 (610/1452, 42.0%) and p.E635Rfs*15 (280/1452, 19.3%). These frequencies are consistent with those reported in myeloid malignancies; Schnittger et al. found that p.G646Wfs*12 accounted for 69 out of 128 *ASXL1* mutations (53.9%), followed by p.E635Rfs*15 (18/128, 14.2%) [36]. These recurrent genetic lesions strongly support the notion that *ASXL1*-CH represents a risk factor for progression to hematologic malignancies [36].

Thus, CH-related *ASXL1* mutations are thought to represent early genetic events contributing to the development of various myeloid malignancies. On the other hand, West et al. reported that *ASXL1* is a second hit following *GATA2* mutations in chronic myelomonocytic leukemia (CMML) [42]. Particularly in CMML, *ASXL1* mutations may play a facilitative role in clonal expansion and tumor progression, rather than the driver mutations.

## Clinical characteristics of *ASXL1*-CH

**Fig. 2:**
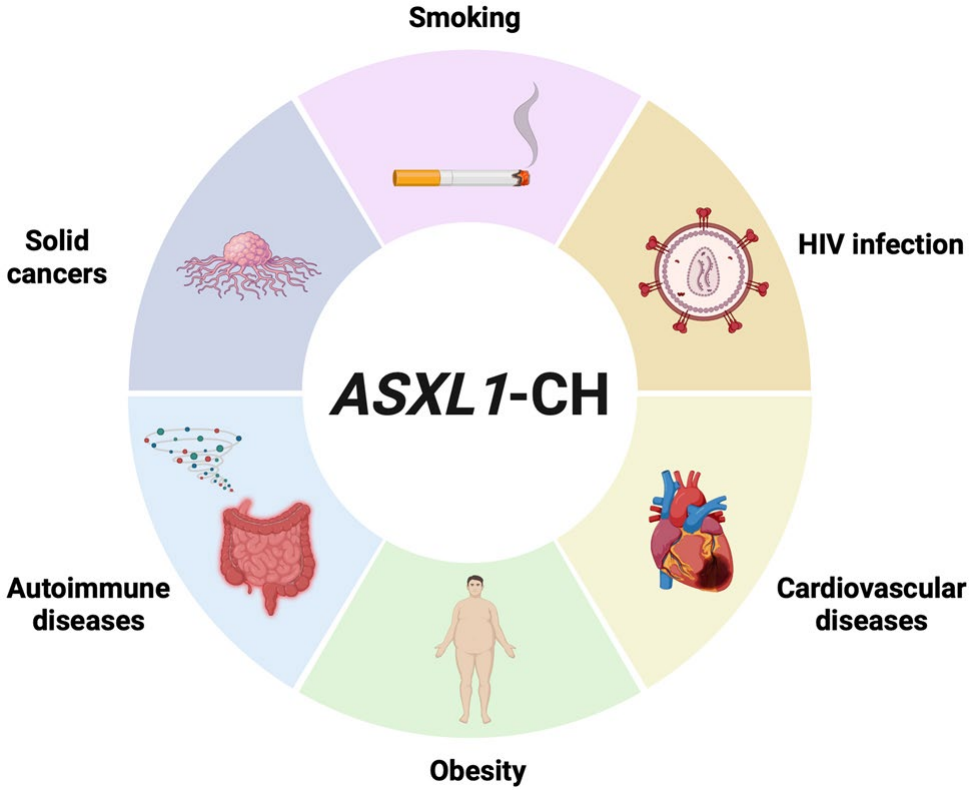
Summary of reported correlations between *ASXL1*-CH and various conditions, including cardiovascular disease, infection, cancers, and autoimmune disorders. All listed conditions share chronic inflammation as a unifying pathological background, potentially mediated by *ASXL1*-CH. *CH* clonal hematopoiesis, *HIV* human immunodeficiency virus. Created in *BioRender. Sato, N. (2025)* https://BioRender.com/fai2raa

In the past decade, an increasing number of epidemiological studies have demonstrated a correlation between CH and age-related diseases that are known to be exacerbated by chronic inflammation. Although the oncogenic roles of *ASXL1* mutations in hematological malignancies are well documented and have been addressed in prior reviews [43, 44], in the following sections, we focus on emerging evidence linking *ASXL1*-mutated CH with lifestyle-associated factors and non-malignant, non-hematological diseases, an area that remains underexplored (Fig. 2).

### CVD and inflammatory signature

Increasing epidemiological evidence suggests a robust relationship between CH and CVD [5, 45]. Similar to *DNMT3A*-CH and *TET2*-CH, *ASXL1*-CH has also been linked to a higher risk of CVD, potentially through pro-inflammatory mechanisms. Bick et al. investigated the associations between CH driver genes and blood cell, lipid, and inflammatory traits in a cohort of 4,229 individuals with CH [20]. In patients with atherosclerosis and CH, interleukin-6 (IL-6) levels were elevated regardless of the specific driver mutation, whereas high-sensitivity C-reactive protein (hsCRP) levels were significantly increased only in those with *ASXL1*-CH. In the same study, *ASXL1*-CH clone sizes in peripheral blood were larger than those of *DNMT3A*-CH or *TET2*-CH. Driver gene-specific inflammatory signatures were also identified: *TET2*-CH was associated with elevated serum IL-1β, while *JAK2*-CH and *SF3B1*-CH correlated with increased IL-18 levels. Interestingly, *ASXL1*-CH was not associated with elevations in IL-1β nor IL-18, despite its strong correlation with hsCRP elevation. Individuals with *ASXL1*-CH also exhibited higher neutrophil and monocyte counts and significantly increased levels of lipoprotein-associated phospholipase A2 (Lp-PLA2), an established independent risk factor for coronary artery disease. Since atherosclerosis is increasingly recognized as a chronic inflammatory disease, these findings support the hypothesis that CH driver gene–specific pro-inflammatory states may contribute to CVD pathogenesis via distinct mechanisms.

Beyond atherosclerosis, chronic systemic and myocardial inflammation are also implicated in the pathogenesis of atrial fibrillation (AF), contributing to structural remodeling, electrical instability, and myocardial fibrosis [46]. In a large cohort study involving 199,982 participants, Saadatagah et al. found that *TET2*-CH and *ASXL1*-CH, but not any CH or *DNMT3A*-CH, were associated with elevated IL-6 levels, indices of cardiac remodeling, and increased risk for AF [47]. These findings further support the notion that *ASXL1*-CH promotes cardiovascular pathology through inflammatory pathways.

Considering the link between CH and CVD via inflammation, immunomodulatory therapy may offer a promising strategy. A sub-analysis of the CANTOS (Canakinumab Anti-inflammatory Thrombosis Outcomes Study) trial demonstrated that anti-IL-1β therapy (canakinumab) significantly reduced cardiovascular events in patients with *TET2*-CH but not in those with CH driven by other mutations, including *ASXL1 *[47]. These results highlight not only the gene-specific inflammatory milieu associated with CH but also the need for tailored therapeutic strategies based on the CH driver mutation.

### Correlation with smoking

Smoking has also been associated with CH, and this appears to be driver gene-specific, particularly for individuals with *ASXL1* driver mutations [48, 49]. Dawoud et al. demonstrated that CH has been associated with smoking, with *ASXL1* mutations showing strong correlations not only with current smoking but also with past smoking history [48]. Indeed, 69% of participants with *ASXL1* mutations were past or current smokers. Recently, Sullivan et al. assessed the impact of cigarettes vs. E-cigarettes on in vivo *Asxl1*^−/−^ cell expansion using competitive transplantation into mice [50]. Although the percentage of *Asxl1*^−/−^ in peripheral blood fell from 60 to 40%, when plated in methylcellulose, splenocytes from the no-smoking group and the E-cigarette substitution group of mice had a 30% decrease in colony proliferation compared to that from the smoking group of mice. Smoking is likely to be associated with expansion of *ASXL1*-CH; however, whethersmoking directly induces CH via gene mutation or why only *ASXL1* mutations have a strong association with smoking remains elusive.

### Correlation with HIV infection

Regarding *DNMT3A*-CH and *TET2*-CH, it has been hypothesized that chronic infections may facilitate the expansion of small clones by altering the bone marrow niche to favor their growth [51, 52]. Human immunodeficiency viruses (HIV) are retroviruses that attack the immune system. Once an individual is infected with this virus, the immune system cannot eliminate it, leading to lifelong infection and chronic inflammation by sustained immune activation [53]. Given the links between CH and chronic inflammation with HIV, several independent studies recently examined the prevalence of CH in individuals living with HIV. Dharan et al. performed targeted amplicon sequencing of CH driver genes in the blood of 202 HIV-positive and 226 HIV-negative patients over the age of 55 years [54]. They found that HIV-positive (HIV^pos^) individuals had 1.68 times the frequency of CH as those who were HIV-negative (HIV^neg^). As the main genes observed in other studies of CH in the general population, the mutations of *DNMT3A* (47.4%), *TET2* (20.0%), and *ASXL1* (13.3%) were identified in HIV-positive individuals. Among the mutated genes, *ASXL1* mutations appeared to be overrepresented in HIV-positive patients compared to HIV-negative participants (*DNMT3A*: 27/52 in HIV^neg^, 37/83 in HIV^pos^; *TET2*: 11/52 in HIV^neg^, 16/83 in HIV^pos^; *ASXL1*: 3/52 in HIV^neg^, 15/83 in HIV^pos^, *P* = 0.004). Another recent study of individuals older than 55 years of age with HIV by Knudsen et al. identified that more than one in four had CH, and the three most mutated genes were *DNMT3A*, *TET2*, and *ASXL1*, accounting for 49, 25, and 16% of mutations, respectively [55]. Further, a study by Bick et al. also examined the prevalence of driver gene-mediated CH in individuals living with HIV, comparing with individuals of a larger population-based cohort, and demonstrated that people living with HIV have a two-fold increase in CH prevalence and that *ASXL1* is the most commonly mutated CH-associated gene [56]. A caveat of this study was that the HIV-positive cohort was a separate cohort from the controls, as the author mentioned, and that several G645Vfs requiring attention to sequencing artifacts were included in *ASXL1* mutations in the cohort. Although HIV infection seems to be associated with a higher prevalence of CH, it remains controversial whether specific CH-related gene mutations, particularly for *ASXL1*, are associated with HIV infection. Chronic infections such as HIV infection may act as a facilitation for the expansion of *ASXL1*-CH clones, as aforementioned hypothesis with *DNMT3A*- and *TET2*-CH. Regardless, the study using larger cohorts of individuals should be performed to uncover the relationship between HIV and CH.

### Correlation with obesity

Using exome sequencing and clinical data of 47,466 individuals with validated CH in the UK Biobank, Pasupuleti et al. demonstrated that the prevalence of CH increased with a higher waist-to-hip ratio (WHR) [57]. They also examined The Cancer Genome Atlas (TCGA) database to determine whether CH was associated with patients with non-hematological cancer with a higher body mass index (BMI) (> 30 kg/m^2^) versus a lower BMI (≦25 kg/m^2^) and found that all analyzed CH genes had a higher mutation rate in patients with a high BMI than in those with a low BMI among all six cancer types examined: bladder cancer, colon adenocarcinoma, cervical squamous cell carcinoma/endocervical adenocarcinoma, rectum adenocarcinoma, skin cutaneous melanoma, and uterine corpus endometrial carcinoma. *ASXL1* mutations were significantly higher in patients with high BMIs in colon adenocarcinoma and tended to be higher in patients with high BMIs in uterine corpus endometrial carcinoma compared with patients with low BMIs. Further, they performed experiments using mouse models of obesity with *Ob*/*Ob* mice and showed an accelerated expansion of hematopoietic stem and progenitor cells (HSPCs) harboring *Tet2*, *Dnmt3a*, *Asxl1*, or *Jak2* mutation. In addition, we also found that a high-fat diet promotes the expansion of blood cells with mutant Asxl1 rather than a control diet in a competitive repopulation assay [58]. Recently, Uddin et al. longitudinally analyzed 4187 participants over a median follow-up of 21 years and found a nominal association between dyslipidemia and *ASXL1*-CH [59]. These studies suggest that the mild chronic inflammation caused by dyslipidemia promotes the development of CH, including *ASXL1*-CH.

### *ASXL1*-CH and autoimmune conditions

As well as chronic infection and obesity, autoimmune diseases also cause chronic inflammation. It has been proposed that the inflammatory environment may accelerate the expansion of certain mutant clones [60, 61]. A study by Hecker et al. performed analyses in a cohort of 200 patients undergoing hip replacement for osteoarthritis and indicated an association between CH and an increased incidence of autoimmune diseases [10]. Moreover, a connection has been documented between CH and inflammatory bowel diseases, such as ulcerative colitis [7, 62]. Cumbo et al. performed a targeted next-generation sequencing analysis in a cohort of 13 patients with IBD associated with hematological malignancies and observed that 11 patients (85%) harbor one or more mutations in CH-associated genes; *DNMT3A* was the most frequently mutated gene, followed by *ASXL1* and *JAK2 *[7].

Although CH-associated mutations have primarily been studied in myeloid malignancies, recent studies have reported *ASXL1* mutations in T-cell–mediated conditions such as aplastic anemia (AA) [14] and large granular lymphocytic leukemia (LGLL) [15]. Notably, Kawashima et al. found that *ASXL1* mutations were frequently detected in LGLL and T-cell clones of uncertain significance (TCUS), while *DNMT3A* mutations, most observed in general CH cohorts, were less frequent in LGLL [15]. Given that CH originates from HSCs, it is plausible that mutations such as those in ASXL1 may influence both myeloid and lymphoid lineages, potentially altering immune cell behavior. However, whether specific CH-associated mutations contribute directly to the development of autoimmune diseases remains to be elucidated and warrants further investigation.

Thus, epidemiological and clinical studies suggest a bidirectional relationship: chronic inflammation may promote the clonal expansion of hematopoietic cells, while CH itself can contribute to systemic inflammation and related diseases. These observations have prompted growing interest in elucidating the molecular mechanisms underlying this interplay.

## Roles of *ASXL1* as an epigenetic regulator

*ASXL1* is identified as one of the human homologues of the Drosophila *Asx* gene [63]. In Drosophila, expression of genes required for somitogenesis during the embryonic stage is regulated by both Trithorax group (TrxG) and Polycomb group (PcG) complexes, having the role of an epigenetic regulator [64]. The PcG complex is divided into two groups: Polycomb repressive complex 1 (PRC1) and Polycomb repressive complex 2 (PRC2), which respectively ubiquitinates H2A at lysine 119 (H2AK119Ub) and trimethylates H3K27 (H3K27me3), resulting in transcriptional inactivation. The TrxG complex trimethylates H3K4, resulting in transcriptional activation. Deletion of *Asx* in Drosophila exhibits TrxG- and PcG-deficient phenotypes [49], suggesting that *Asx* is indispensable for histone modifications by TrxG and PcG proteins. Similar to Drosophila Asx, recent studies uncovered the relationship between *ASXL1* and histone modifications in mammals. The human *ASXL1* gene is located on chromosome 20q11 and encodes a 1541 amino acid protein [63]. *ASXL1* regulates gene expression through interactions with multiple proteins, such as BAP1 [65], BMI1 [66], BRD4 [67], EZH2 [68, 69], and FOXK1 [70]. *ASXL1* has an N-terminal ASXN domain, an ASX homology (ASXH) domain at the N-terminal region, and a PHD finger at the C-terminal region. The ASXN domain is structurally similar to the Forkhead-box (FOX) domain and is predicted to be essential for the DNA-binding ability [71]. The ASXH domain is highly conserved from Drosophila to mammals and is a deubiquitinase adaptor domain (DEUBAD) because this domain binds a deubiquitinase BAP1 [72].

The C-terminal PHD domain is a putative histone-binding module and recognizes different histone modification subtypes, such as unmethylated H3K4 (H3K4me0) and trimethylated H3K4 (H3K4me3) [73, 74]. In relation to this, Wang et al. reported that *ASXL1* loss in mice causes reduced global levels of H3K4me3 [75].

While canonical PHD fingers typically recognize H3K4me3 through a conserved aromatic cage, the PHD domain of *ASXL1* appears structurally atypical and lacks several of the key residues found in high-affinity methyl-lysine readers [76, 77]. Structural and biochemical analyses suggest that ASXL1-PHD finger binds H3K4me3 with weaker affinity than classical PHD domains or may recognize alternative chromatin-associated signals [75, 76]. Nevertheless, *ASXL1* is functionally linked to the maintenance of H3K4me3, likely through its interaction with the OGT–HCFC1 complex and MLL family methyltransferases [78].

It is therefore plausible that the PHD finger of *ASXL1* serves not as a canonical high-affinity reader, but rather as a scaffold or targeting module, stabilizing methyltransferase complexes at H3K4 me3-enriched regions. Such a mechanism would parallel known epigenetic feedback systems, including PRC2, where reader domains (e.g., EED) bind the very mark they help establish, reinforcing chromatin states through a read–write loop [79]. This mechanism reconciles the dual association of *ASXL1* with both the promotion and recognition of H3K4me3 and supports a model in which its PHD finger contributes to transcriptionally active chromatin maintenance [78].

Collectively, *ASXL1* regulates gene expression via various histone modifications, such as H3K27me3, H2AK119Ub, and H3K4me3, through interactions with multiple proteins.

## Novel findings about the functions of *ASXL1*

**Fig. 3:**
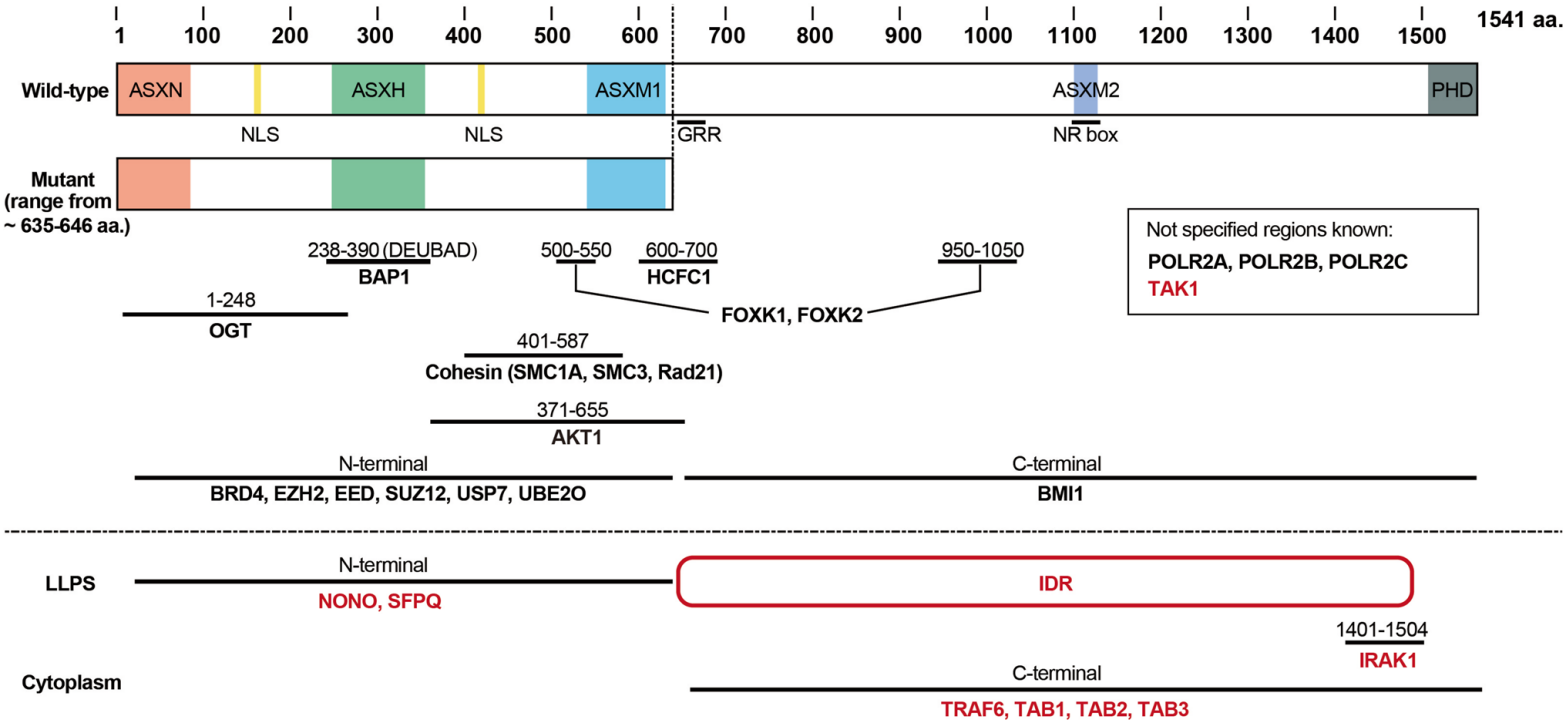
Schematic representation of the interacting proteins of wild-type ASXL1 and C-terminally truncated mutant ASXL1. The ASXL1-interacting proteins newly identified are red colored. *NLS* = nuclear localization signal; *LLPS = *liquid-liquid phase separation; *DEUBAD = *deubiquitinase adaptor domain; *IDR =* intrinsically disordered regions

*ASXL1* is mainly known to play roles as the epigenetic regulator in the nucleus, however, emerging reports have indicated other functions with interacting proteins (Fig. 3). Yamamoto et al. demonstrated that wild-type *ASXL1* is involved in liquid–liquid phase separation (LLPS), as in the paraspeckle formation, increasing NONO-NEAT1 interactions through the C-terminal intrinsically disordered region (IDR) [80]. In contrast, the mutant *ASXL1* lacking IDR did not support the interaction of paraspeckle components, disrupting paraspeckles and RNA splicing in HSPCs. Otherwise, Latacz and colleagues reported that *ASXL1* truncations form strong phase-separated condensates, whereas the wild-type does not [81]. Although further research is still needed for the contrasting results, these studies should provide a novel insight into the mechanisms of dysregulated gene expression by which is not collateral in histone modification in HSPCs harboring *ASXL1* mutations.

Furthermore, several reports showed localization of *ASXL1* both in the cytoplasm and nucleus, whereas C-terminal truncated *ASXL1* mutants were predominantly present in the nucleus [58, 70, 82, 83]. From our group, Fujino and his colleagues reported that C-terminally truncated mutant *ASXL1* cooperates with BAP1 to deubiquitinate and activate AKT in both the cytoplasm and the nucleus [82]. Additionally, we recently reported that full-length *ASXL1* interacts with IRAK1 via its C-terminal region, inhibiting IRAK1-TAK1 signaling, whereas the mutant *ASXL1* does not [58]. An IRAK1/4 inhibitor ameliorated the *ASXL1* mutant-driven increase of myeloid cells in the peripheral blood, resulting in the reduction of the atherosclerotic lesion size. Further studies are warranted to elucidate the role of novel *ASXL1* functions in biological processes and the pathogenesis of CH and hematopoietic disorders.

## Studies using in vivo models of *ASXL1* mutations

A series of early studies established *Asxl1* knockout mice to reveal the role of *ASXL1* in hematopoiesis and observed that those mice recapitulate human MDS-like pathologies [75, 84, 85]. However, as described above, *ASXL1* mutations are always heterozygous, and the mutant *ASXL1* transcripts are thought to produce a C-terminally truncated protein escaping nonsense-mediated decay [86]. The following studies indicated that truncating mutations in *ASXL1* can lead to gain-of-function by enhancing the enzymatic activity of BAP1, promoting H2AK119 deubiquitination [65, 87, 88]. Thus, whether truncating mutations of *ASXL1* result in gain- or loss-of-function or confer dominant-negative activity in vivo remains controversial. Establishing in vivo models for *ASXL1* mutations is pivotal to revealing the biological functions and their contribution to the underlying mechanisms of diseases. Next, we introduce several in vivo models developed by different research groups (Table 1), which allow for investigating the impact of *ASXL1* alteration in hematopoiesis and myeloid malignancies. The reader is referred to each reference for a more detailed discussion of this mechanism. The present review summarizes only the key aspects relevant to our focus.

**Table 1:** Overview of in vivo models of *Asxl1* mutations

Mouse model		Experimental settings	HSC phenotype	Disease phenotype	Histone modification	References
Constitutive knockout	*Asxl1*^tm1Bc/tm1Bc^	primary	Slight reduction of GM and GEMM colony-forming ability	Reduced T cells in thymus, Reduced B cells in BM, splenomegaly with increased myeloid cells	No data	[84]
	*Asxl1*^−/−^ and *Asxl1*^±^	primary/transplantation	Decreased LSK in homo	MDS-like disease	Reduced H3K4me3 and H3K27me3	[63]
Conditional knockout	*Asxl1*^fl/fl^;Mx1-Cre/Vav1-Cre	primary/transplantation/ induction	Increased LT-HSC and LSK	Leukopenia and amenia, dysplasia	Reduced H3K27me3	[85]
Constitutive knock-in into *Asxl1* locus	*Asxl1*^G643fs^	primary/transplantation	Decreased repopulation ability	Non observed (18 mo)	Changes in distribution of H3K27me3	[89]
	*Asxl1*^G643fs^	primary/transplantation	Decreased LT-HSC and LSK	MDS/MPN-like disease	Reduced H2AK119Ub	[81]
*Vav1* promoter-driven transgenic	*Asxl1*^*Y588X*^	primary/transplantation	Increased ST-HSC and LSK	AML, MPN, and MDS-like disease	Increased levels of H3K112Ac and H3K27Ac	[67]
Overexpression	human *ASXL1* (wild-type, G646Wfs or E635Rfs)	overexpression/transplantation	Not evaluated	AML, MDS-like disease	Reduced H3K27me3	[69]
Conditional knock-in into *Rosa26* locus	*Asxl1*^E635Rfs^;Vav1-cre	primary/transplantation	Decreased LT-HSCs (4-mo old), decreased repopulation ability	Non observed (18 mo)	Reduced H3K4me3, H2AK119Ub, H3K27me3	[90]
	*Asxl1*^E635Rfs^;Vav1-cre or Mx1-Cre	primary/transplantation/induction	Increased LT-HSCs with aging (aged Vav1-cre, Mx1-cre)	Mild anemia, myeloid skewing, and increased platelets in old age	Reduced H3K4me3 and H2AK119Ub	[82]

Zebrafish model		gRNA sequence	HSC phenotype	Disease phenotype	Histone modification	References
CRISPR/Cas9	*asxl1*^e12^	GGGATACCGGATCGCTCCAGCGG	Increased HSPCs expressing anti-inflammatory genes	Clonal expansion with elevated pro-inflammatory cytokines	No data	[93]
CRISPR/Cas9	*asxl1*^e12 (−22)^	GGGTGTGGATGTCATCAGGACAT	Not evaluated	AML, CMML, and MDS-like disease	Decreased global H2AK119ub and H3K4me3	[94]

Mouse model of CVD/Obeity		Experimental settings/gRNA sequence	HSC phenotype	Disease phenotype	Changes of cytokines	References
Constitutive knock-in into Asxl1 locus	Asxl1G646Wfs*/+	transplatation/LAD ligation /Ang II infusion	No significant differences were observed	Accelerated heart failure	Increased expression of Il1b and Il6 in macrophages	[95]
CRISPR/Cas9	Asxl1G623*	transplatation into Ldlr–/–mice AGTGGTAACCTCTCGCCCCTCGG	No data	No change of atherosclerosis No hematopoietic diseases were observed	Increased IL-6 and IL-10 secretion and decreased TNF secretion by macrophages	[96]
Conditional knockout	Asxl1±;Mx1-Cre	transplantation into Ob/Ob mice	Increased LT-HSC and LSK	Upregulation of proinflammatory cytokines	Increaed serum cytokines and chemokines; IL-6, IL-1α, IL-1β, IL-10, TNF-α, GM-CSF, CCL-2 and CXCL-9	[57]
Conditional knock-in into Rosa26 locus	Asxl1E635Rfs;Vav-cre or Mx1-Cre	transplatation into Ldlr–/–mice, transplantation into B6 mice followed by LFD or HFD feeding	Increased LT-HSC and LSK	Accelerated atherosclerosis	Increased serum IL-6 levels in mice Increased expression of Il6 in macrophages	[58]

The studies using the *Asxl1* knockout mouse models showed development into MDS-like diseases, except for that of the study by Fisher et al. [75, 84, 85]. Constitutive loss of *Asxl1* results in developmental abnormalities, recapitulating the clinical features of the human Bohring-Opitz syndrome. Hematopoietic-specific deletion of *Asxl1* results in the abnormal self-renewal capacity of HSCs exhibiting the increased expression of posterior Hoxa genes and in multilineage cytopenia and dysplasia. While these results underscore the importance of *ASXL1* in regulating gene expression during normal hematopoiesis, the relatively rapid disease course, although species differences must be considered, seems somewhat unexpected for an isolated *ASXL1* mutation.

On the other hand, knock-in mouse models of *ASXL1* mutations showed relatively mild phenotypes. As described above, c.1934dupG; p.G646Wfs*12 is the most common *ASXL1* mutation both in human CH and myeloid malignancies [36, 41]. Hsu and colleagues generated a knock-in mouse harboring a G643fs mutation of the *Asxl1* locus, which mimics the human mutation. These mice did not develop any blood malignancies [89]. Similarly, our group generated a conditional *Asxl1*-mutant (mimicking *ASXL1* E635RfsX15) knock-in (Rosa 26 locus) mouse model and found that the old mice with the *Asxl1*-mutant showed myeloid skewing, thrombocytosis, and mild anemia, without developing MDS or AML [82, 90]. Unlike the knockout mouse models, these results indicate that CH-related *ASXL1* mutation alone is not sufficient for the development of blood malignancies like human *ASXL1*-CH. However, Uni et al. also generated a knock-in mouse model with G643fs in the *Asxl1* locus and found that their mice developed the human MDS or myeloproliferative neoplasm-like diseases after long latency [66]. Another, Yang and colleagues generated the Vav1 promoter-driven *Asxl1*^Y588X^ transgenic mouse model and found the development of hematological malignancies [67]. Notably, Inoue et al. reported the development of myelodysplastic syndrome in a transplantation model employing enforced expression of mutant *ASXL1* [69]. In light of these findings, it is conceivable that phenotypic variation may arise from differences in experimental design or the expression levels of the mutant allele. Moreover, the *Asxl1* mutations change epigenetic regulations and may cause genome instability, which may resultin an increased potential for additional acquired mutations.

Focusing on histone modifications, knockout mouse models primarily exhibit a reduction in H3K27me3, whereas knock-in models additionally show a decrease in H2AK119Ub. This discrepancy is thought to reflect the enhanced deubiquitinase activity of BAP1 mediated by mutant *ASXL1* [65, 91, 92].

In recent years, CRISPR–Cas9–mediated genome editing has enabled a more straightforward approach to introducing mutations. Utilizing zebrafish models, two independent groups performed experiments introducing mutations in HSCs targeting the last exon of *asxl1* [93, 94]. Although phenotypic differences have been reported across models, they consistently demonstrate clonal expansion of HSCs harboring *asxl1* mutations. Interestingly, Avagyan et al. reported that differentiated macrophages derived from *asxl1*-mutant HSPCs exhibit a pro-inflammatory gene expression profile, whereas HSPCs maintain a predominantly anti-inflammatory transcriptional signature, resulting in conferring them a selective advantage [93]. The experimental approach using CRISPR-Cas9 will increase because of its ease of handling, however, whether CRISPR–Cas9–induced mutations lead to the expression of a C-terminally truncated *ASXL1* protein, as observed in knock-in models, remains unclear, as current reports have not provided evidence on this point.

In summary, the functional consequences of *ASXL1* mutations appear to be diverse and context-dependent. Knockout and knock-in mouse models have consistently demonstrated that loss of *ASXL1* function leads to reductions in histone modifications such as H3K27me3 and H3K4me3, supporting a loss-of-function mechanism in epigenetic regulation, while reduction of H2AK119ub is not altered in knockout models. Notably, these truncating mutations frequently preserve the N-terminal domains responsible for complex formation with factors such as BAP1 or OGT–HCFC1, suggesting the potential for dominant-negative effects. While the extent to which gain-of-function activity contributes to disease remains uncertain, these observations imply that truncated *ASXL1* may interfere with normal epigenetic control in a multifaceted manner, depending on the cellular context and cooperating mutations.

## Studies of age-related diseases using in vivo models of *ASXL1* mutations

Epidemiological studies have brought increasing attention to the association between CH and various age-related diseases, particularly CVD. Experimental approaches for the underlying mechanism of the CH-disease association have primarily been driven by advances in research, mainly on *TET2* and *DNMT3A*. Three independent groups, including ours, have recently investigated the association of *ASXL1* mutations and CVD using distinct murine models for CVD, which is the most closely correlated with CH (Table 1). Min et al. employed an *ASXL1* knockout model in the context of pressure overload-induced or ischemia-induced heart failure and demonstrated that hematopoiesis driven by *ASXL1*-deficient cells promoted myocardial fibrosis and impaired cardiac function [95]. In this study, macrophages lacking *ASXL1* exhibited elevated expression of proinflammatory cytokines, including *Il1b* and *Il6*. Yu et al. employed a CRISPR–Cas9-based approach to editing the last exon of *Asxl1* in murine bone marrow cells (*Asxl1*-G623*), mirroring prior studies in zebrafish, and investigated the impact on inflammasome activity in bone marrow-derived macrophages (BMDMs) as well as atherosclerotic lesion formation [96]. Their findings demonstrated that BMDMs exhibited an enhanced inflammatory response to AIM2 agonism, associated with an upregulated DNA damage response, leading to increased secretion of IL-1β and IL-10. However, transplantation of the edited LT-HSCs into *Ldlr*^−/−^ atherosclerosis-prone mice did not result in clonal expansion relative to wild-type hematopoietic cells, nor did it lead to increased atherosclerotic burden.

In contrast, our group utilized a knock-in mouse model carrying a mutation (mimicking *ASXL1*^E635R^ in humans) and observed that following transplantation into *Ldlr*^−/−^ mice, *Asxl1*-mutant clones displayed a clear competitive advantage and promoted the development of atherosclerotic lesions [58]. BMDMs derived from mutant *Asxl1* knock-in mice secreted higher levels of IL-6, and single-cell transcriptomic analysis of the atherosclerotic plaques revealed expansion of both granulocytic and macrophage compartments. Moreover, monocyte/macrophage populations exhibited upregulated activity of the Toll-like receptor–NF-κB signaling pathway.

Another, Pasupuleti et al. and our group demonstrated that hematopoietic cells with *Asxl1* mutation induced after transplantation expanded in obesity-model mice [57, 58]. Considering these findings, *ASXL1*-CH is likely to expand with acceleration in atherogenic conditions, including dyslipidemia and obesity.

What makes the difference among the studies in the fitness advantage of *Asxl1*-mutated clones? As noted by Yu et al. as a limitation of their study [96], and supported by findings from Fujino et al. [82], *Asxl1* mutations can impair engraftment capacity and hematopoietic reconstitution in transplantation settings while conferring a proliferative advantage in inducible models. These observations underscore the importance of carefully selecting disease modeling strategies, suggesting that either pre-induction of *Asxl1* mutations prior to transplantation should be avoided, or non-competitive transplantation approaches should be considered to minimize the confounding effects of impaired reconstitution [43, 44].

On the association between CH and solid cancers, our group demonstrated that T-cell-specific expression of mutant *Asxl1* accelerated the growth of melanoma, lung, and colon cancer cells in allogeneic xenograft models [97]. Mutant *Asxl1* induced aberrant intrathymic T-cell development, decreased CD4/CD8 ratio, and naïve-memory imbalance, indicating that *ASXL1*-CH contributes to creating a protumor microenvironment for solid tumors by impairing T-cell growth and function.

Considering the impact of *ASXL1* mutations on both the function and composition of immune cells, developing appropriate in vivo models is essential to elucidate the mechanisms linking ASXL1-CH with age-related diseases. However, current disease models remain limited, and further refinement of experimental systems is required to specifically explore how *ASXL1*-CH contributes to the pathogenesis of the diverse disorders discussed earlier in this review.

## Concluding remarks

Compared to the accumulation of somatic mutations in normal tissues during aging, genetic alterations observed in CH are disproportionately enriched in epigenetic regulators. From the perspective of multistep carcinogenesis, CH is frequently discussed in terms of its mutational origins, age-dependent dynamics, and progression to overt hematological malignancies. However, what is particularly noteworthy is the growing body of epidemiological evidence linking CH, especially in cases without overt hematological symptoms, to a broad spectrum of age-related diseases, many of which appear unrelated to blood cells at first glance.

Among these, *TET2*-CH and *DNMT3A*-CH have been extensively studied in efforts to elucidate the mechanisms connecting CH to age-associated conditions. In contrast, in vivo modeling of other mutations, such as those in *ASXL1* or splicing factors, has been complicated by issues related to insufficient clonal advantage in transplantation settings. In the case of *ASXL1* mutations in particular, special attention must be paid to potential sequencing artifacts at known hotspot loci, and to the fact that the pathogenic mechanisms likely involve gain-of-function or dominant-negative effects rather than simple gene loss, making the development of appropriate in vivo-disease models inherently challenging.

Nevertheless, as highlighted through the various in vivo models reviewed here, one emerging conclusion is that any transplantation-based approach must carefully account for the impact of *ASXL1* mutations on engraftment capacity. Notably, it is of particular interest that pro-atherogenic conditions—possibly including obesity—appear to overcome the previously reported competitive disadvantage associated with *ASXL1* mutations.

To further clarify the wide-ranging consequences of *ASXL1*-CH, including its potential links to aging-related accelerants such as smoking, obesity, and HIV infection, as well as its associations with autoimmune diseases, cardiovascular pathology, and solid tumors, both large-scale longitudinal epidemiological studies and experimentally validated in vivo models are needed. Comprehensive multi-omics approaches will be essential to delineate the lineage-specific impacts of *ASXL1*-mutant hematopoiesis.

## Data Availability

The dataset of localization of *ASXL1* mutations in individuals with CH by Vlasschaert et al., described in Fig. 1, is available on the Blood website at 10.1182/BLOOD.2022018825. [41]
